# Hormonal Changes in High-Level Aerobic Male Athletes during a Sports Season

**DOI:** 10.3390/ijerph17165833

**Published:** 2020-08-12

**Authors:** Javier Alves, Víctor Toro, Gema Barrientos, Ignacio Bartolomé, Diego Muñoz, Marcos Maynar

**Affiliations:** 1 Department of Sport Science, Faculty of Education, Pontifical University of Salamanca, C/Henry Collet, 52–70, CP: 37007 Salamanca, Spain; fjalvesva@upsa.es; 2 Department of Physiology, Faculty of Sports Science Faculty, University of Extremadura, University Avenue, s/n CP: 10003 Cáceres, Spain; vtororom@alumnos.unex.es (V.T.); ignbs.1991@gmail.com (I.B.); diegomun@unex.es (D.M.); mmaynar@unex.es (M.M.)

**Keywords:** hormones, LH, testosterone, cortisol, insulin, athletes

## Abstract

The aim of this study was to determine the possible changes in plasma of several hormones such as Luteinizing Hormone, Testosterone, Cortisol and Insulin in endurance runners during the sports season. Twenty-one high-level male endurance runners (22 ± 3.2 years, 1.77 ± 0.05 m) participated in the study. Basal plasma hormones were measured at four moments during the season (initial, 3, 6 and 9 months), and were analyzed using ELISA (enzyme-linked immunosorbent assay). Testosterone and Luteinizing Hormone (LH) suffered very significant decreases (*p* < 0.01) at 3 months compared with the beginning and an increase (*p* < 0.05) at 6 and 9 months compared with 3 months. Insulin level was significantly lower (*p* < 0.05) at 3, 6 and 9 months compared with the initial test. Insulin and cortisol were associated inversely (r = 0.363; β = −0.577; *p* = 0.017) and positively (r = 0.202; β = 0.310; *p* = 0.043), respectively, with the amount of km per week performed by the runners. There was a significant association between km covered at a higher intensity than the anaerobic threshold and I (r = 0.580; β = −0.442; *p* = 0.000). Our findings indicate that testosterone, LH and insulin were more sensitive to changes in training volume and intensity than cortisol in high-level endurance runners. Basal testosterone and LH concentrations decrease in athletes who perform a high volume of aerobic km in situations of low energy availability.

## 1. Introduction

Endurance athletes modulate the volume and intensity of their constant training throughout the sports season in order to produce adaptations and achieve their best performance in previously established competitive periods [1]. This exercise causes stress in the organism that induces important changes in the endocrine system to recover the initial homeostasis [2].

Cortisol (C) is the main glucocorticoid of the organism, its secretion is produced in the adrenal glands and it is controlled through a negative feedback mechanism by the hypothalamus–pituitary–adrenal axis [3]. It is a hormone modulated by circadian rhythms, but factors such as mental stress, dehydration or food can alter its production [4]. In endurance activities, its blood values increase, as its catabolic function contributes to maintaining adequate energy levels through protein degradation, hydrolysis of triglycerides and even adding additional energy from carbohydrates through gluconeogenesis in the liver [5]. At the end of physical activity, the concentrations of this hormone begin to decrease, and it may take up to 48 h to recover its basal values after maximum effort [6].

Testosterone (T) is an anabolic hormone that participates in multiple physiological functions, intervenes in muscle protein synthesis, stimulates bone remodeling and erythropoiesis [7] and regulates the function of lactate transporter proteins thus promoting lactate oxidation as a fuel during exercise [8]. T is secreted by the Leydig cells in the testicles and its concentration in blood is controlled by the hypothalamic–pituitary–testicular (HPT) axis [9]. It has been reported that athletes who undergo long-term continuous training may have reduced levels of chronic basal T, status defined as “Exercise-Hipogonadal Male Condition” [10]. Previous studies have shown that its low levels would be caused by the negative relationship between C and T [11,12]. Hackney, Szczepanowska and Viru have hypothesized whether this inhibition would be caused by a dysfunction of the axis at the peripheral level, through direct inhibition of the Leydig cells in the testicles, or at the central level, reducing the release of Luteinizing Hormone (LH) in the pituitary that would affect the production of T at the testicular level [13].

LH is secreted in the anterior pituitary gland. LH is part of a pathway comprising the hypothalamus, pituitary gland, and gonads [14]. The release of LH is stimulated by gonadotropin-releasing hormone (GnRH) [15]. After acute physical exercise, LH usually decreases [16,17]. During the training phases, a reduction in LH secretion was found in runners [18].

The T/C ratio is a variable that relates to the anabolic/catabolic balance in athletes and is widely used for monitoring and evaluating the body’s response to chronic exercise-induced stress [19]. Authors such as Meeusen et al. [20] think that the ratio cannot be used as a means of control since they reported studies where decreases of 30% of the ratio did not always worsen the athletes’ performance.

Another important hormone involved during physical exercise is insulin (I), related to energy balance and blood glucose control [21]. Horton, Grunwald, Lavely and Donahoo reported a decrease in plasma I concentration during exercise, followed by an increase during the hours after exercise to favor glycogen repletion, a decrease in carbohydrate oxidation, and an increase in fat oxidation [22]. Aerobic endurance athletes have lower baseline values and higher insulin sensitivity than sedentary subjects to support fatty acid oxidation [23].

In summary, hormones have been defined as important mediators in the body’s response and adaptations to exercise-induced stress. Their acute responses to different stimuli and their modifications over short periods have been extensively investigated [24,25]. However, few studies have shown hormonal changes in high-level endurance runners during a sports season, so the aim of this study was to determine the baseline values of T, C, LH and I and their changes throughout a sports season where training loads are modulated to obtain several peaks of performance.

## 2. Materials and Methods

### 2.1. Participants

The athletes were studied every three months at four moments during an athletic season. The measures were made during the first week of October, January, April and July. Athletes were informed of the purpose of the study and signed an informed consent form prior to enrolment. A code was assigned to each participant for the collection and treatment of the samples in order to maintain their anonymity. This research was carried out under the Helsinki Declaration ethical guidelines, updated at the World Medical Assembly in Fortaleza (Brazil) in 2013 for research with human subjects, and the Ethics Committee of the University of Extremadura approved the protocol (52/2012).

Twenty-one high-level aerobic male runners (22 ± 3.2 years, 1.77 ± 0.05 m) participated in the present survey, all of them were living in the area of Caceres (Spain), at a latitude of 39° 28’ 35.36” N. Each athlete had at least five years of training experience, and all of them were participants in national and international tournaments (1500 and 5000 m race modalities). All subjects were required to have a stable body weight throughout the sports season (no weight changes >3%). Significant changes in fat mass and fat-free mass (FFM) are associated with circulating T concentrations due to their role in energy metabolism and adipogenesis [26]. The participants did not take regular medication, anti-inflammatory medications or nutritional supplementation during the two weeks prior to the measurements. None of the subjects had taken hormonal medication in the previous year or during the study since any high-level athlete is obliged to conform to drug testing in competition or out of competition.

### 2.2. Nutritional Evaluation

All participants were instructed to complete a 3-day diet record, including one weekend day and two weekdays, on the provided nutritional questionnaire; each participant weighed and indicated the amount in grams of each food consumed. The athletes’ dietary intakes were obtained using a food composition table [27].

### 2.3. Anthropometrics Measurements

Subjects reported to the laboratory after an overnight fast and had to abstain from hard training and/or competition for at least 72 h before testing. The participants’ morphological characteristics were measured in the morning and always at the same time (09:00 a.m.). Body height was measured to the nearest 0.1 cm using a wall-mounted stadiometer (Seca©, Hamburg, Germany), and body weight was measured to the nearest 0.01 kg using calibrated electronic digital scales, (Seca©, Hamburg, Germany) in barefoot conditions. Fat mass and fat-free mass content was estimated from the sum of 6 skinfolds (∑6) (abdominal, suprailiac, tricipital and subscapularis, thigh and calf). Skinfold thicknesses were measured with a Harpenden caliper (Holtain Skinfold Caliper, Crosswell, UK) and converted to % of body fat using the equations of Jackson and Pollock [28]. All measurements were made by the same operator, accredited in kinanthropometric techniques (level 1), in accordance with the International Society for the Advancement of Kinanthropometry (ISAK) recommendations [29].

### 2.4. Exercise Test until Exhaustion

A running test on a treadmill (Powerjoc, UK) equipped with a gas analyzer (Metamax, Cortex Biophysik Gmbh, Germany) and a Polar pulsometer (Polar Vantage M, Norway) was used to evaluate the maximum oxygen uptake (VO_2_ max). All the tests were performed between 10 and 12 a.m. Exercise test consisted of a 10 min warm-up at 10 km/h followed by incremental runs until voluntary exhaustion, starting at 10 km/h and increasing it by 1 km/h every 400 m, with a stable slope of 1%. During the incremental test, VO_2_ max was determined according to the following criteria: the respiratory exchange ratio (RER) had to exceed 1; stabilization in oxygen uptake (VO_2_) together with an increment in carbon dioxide (CO_2_) elimination and in the ventilatory volume (VE), induced by the increases in the test velocity.

After recording the test data, the ventilatory thresholds were determined according to the three-phase model to monitor training [30]. The data were obtained at the aerobic threshold (VT_1_) and the anaerobic threshold (VT_2_) to determine training load intensity.

### 2.5. Training Characteristics

**Figure 1:**
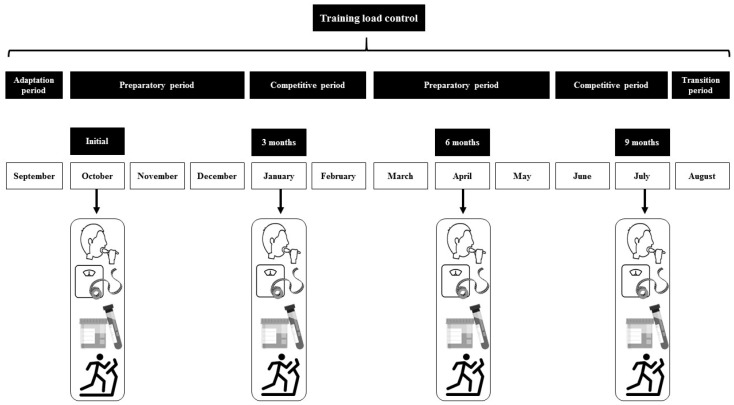
Periodization and testing during the season.

Figure 1 details the timeline of periodization and testing during the season. Athletes had a four-week adaptation before the initial measurement (October), where they performed 85.71 ± 13.62 km per week and a four-week transition period after the second competitive period. The first preparatory period began in October through December and the second one during March through May. Competitive periods were coincident with January and February when the athletes performed cross country competitions (10,000–12,000 m approximately), and the second competitive period was in June-July when they performed track and field competitions between 1500 and 5000 m. A GPS pack equipped with pulsometers (Polar Vantage M. Norway) was used to track the training loads during the season.

Table 1 summarizes training characteristics in the athletes. In addition, they performed two weekly sessions of resistance training during the whole athletics season. In general, the volume of the training was high (3 sets of 8–12 repetitions of whole-body exercises) while the intensity was low-moderate (30–70% of 1RM) depending on the period of the season.

### 2.6. Sample Collection

Always at nine o’clock in the morning, to limit the impact of circadian rhythms on hormonal concentrations, after weighing the participants, ten milliliters of antecubital venous blood was drawn from each participant. Venous blood samples were obtained using EDTA as anticoagulant. Blood was immediately centrifuged at 3000 rpm during 10 min (P-selecta, MEDITRONIC) using a plastic syringe with a stainless-steel needle. The blood sample was collected in a polypropylene tube. Then, the blood sample was centrifuged at 3000 rpm for 15 min at room temperature (23 ± 1 °C) to separate plasma from erythrocytes. Plasma was placed in sterile tubes and stored at −80 °C until use.

### 2.7. Analytical Determination

The hormone determination was carried out using the ELISA (enzyme-linked immunosorbent assay) with an ER-500 (Sinnowa, Germany), using the commercial tests for I, C, T and LH. All hormonal measurements were performed by the same technician and were made with duplicate determination. Between and within coefficients of variation for all assays were less than 10% for all biochemical analyses.

### 2.8. Statistical Analysis

The statistical analysis was carried out with IBM SPSS Statistic software version 21.0 (IBM Co., Armonk, NY, USA). The results are expressed as x ± s, where x is the mean values and s is the standard deviation. All variables used in the study were checked for normality of distribution before the analyses (Kolmogorov–Smirnov tests). The data were analyzed by repeated measurements analysis of variance (ANOVA) with the Bonferroni post hoc test for moment/period as the categorical variable. Partial eta squared (η_p_^2^) was used as an effect size measure of ANOVA. Threshold values for assessing magnitudes of standardized effects were η_p_^2^ ≥ 0.01, η_p_^2^ ≥ 0.06 and η_p_^2^ ≥ 0.14 for small, medium and large, respectively [31]. The equality of variances between the differences was assessed with Mauchly’s test of sphericity. When sphericity was violated, Greenhouse–Geisser corrected *p*-values were used. Simple linear regression analysis was conducted to examine the associations between hormones and km trained per week. A *p* ≤ 0.05 was considered statistically significant.

## 3. Results

Table 2 shows ergospirometric and body composition variables in the runners during the season. In our study, VO_2_ max, VT_2_, RER, heart rate maximum (HRM), fat mass, fat-free mass and ∑6 skinfolds did not show significant changes during the season. Weight suffered significant decreases (*p* < 0.05) at 6 and 9 months compared with initial values.

Nutritional intake of the athletes during the season is shown in Table 3. The athletes followed a diet using established energy and macronutrient guidelines for adequate athletic performance [32]. Energy availability (EA) is defined as the amount of energy intake (kcal day^−1^) −exercise energy expenditure (kcal day^−1^)] /FFM. An appropriate energy balance equates to ≥45 calories per day per kg of FFM (kcal/kg/FFM/d) [33].

Plasmatic concentrations of hormones are shown in Table 4.

**Figure 2:**
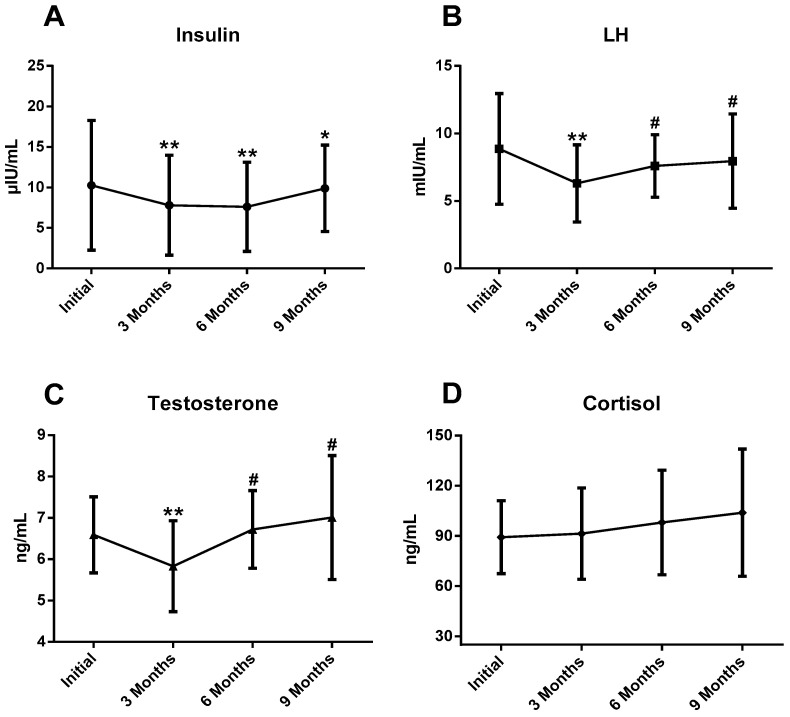
This figure shows the key findings. (**A**) Insulin changes during season; (**B**) LH changes during season; (**C**) testosterone changes during season; (**D**) cortisol changes during season; LH: luteinizing hormone; * *p* < 0.05 differences between initial vs. 3/6/9 months; ** *p* < 0.01 differences between initial vs. 3/6/9 months; # *p* < 0.05 differences between 3 months vs. 6/9 months.

Analysis of the data revealed changes in plasmatic concentrations of total T, LH and I during the sports season. T and LH suffered very significant decreases (*p* < 0.01) at 3 months compared with the initial test, and an increase (*p* < 0.05) at 6 and 9 months compared with 3 months. I concentrations were significantly lower at 3, 6 (*p* < 0.01) and 9 (*p* < 0.05) months compared with the initial test (Figure 2). There were no statistical differences in plasmatic C concentration and T/C ratio during the athletic season.

Simple linear regressions between the plasmatic hormones and km trained are shown in Table 5 and Table 6. Plasmatic concentrations of I and C were inversely (r = 0.363; β = −0.577; *p* = 0.017) and positively (r = 0.202; β = 0.310; *p* = 0.043) associated, respectively, with the amount of km trained per week.

There was a significant association between km trained at a higher intensity than VT_2_ and I (r = −0.580; β = −0.442; *p* = 0.000).

## 4. Discussion

The purpose of our longitudinal study was to observe the changes in plasma basal concentrations of LH, T, C and I in high-level endurance runners, as well as the possible changes that occur during a sports season in relation to the training performed.

VO_2_ max and VT_2_ did not show significant changes during the season in our athletes. High values of VO_2_ max are required in endurance athletes, although it is not a determinant variable among homogeneous groups [34]. Body composition and running economy are other variables related to performance in endurance runners [35,36].

The findings of this research agree with those observed in other studies in which they found that training throughout a sports season produces adaptations in the endocrine system with the aim of improving the athletes’ performance [2]. The basal concentrations of the different hormones in the study showed significant changes during the sports season although they remained within the normal reference values for humans [37,38].

C did not suffer significant changes during the season, since athletes were examined in the laboratory without having carried out intense exercise the previous days. Mäestu, Jürimäe and Jürimäe reported that C did not change when training volume was increased [39]. In our study, C showed a positive association with the volume of km performed per week. Purge, Jürimäe and Jürimäe also observed a significant relation between C and mean training volume in elite male rowers [40]. It is known that during long-term aerobic exercise hypercortisolemia occurs which contributes to maintaining adequate energy levels during training [9]. The C prevents the re-esterification of fatty acids released by the catecholamine-induced lipolysis [41]. The activation of catabolic processes is an essential tool for adaptation in high-stress conditions [42].

Cortisol is usually elevated in energy-deficient conditions. Increases in cortisol circulation have been observed in studies of severe caloric restriction or fasting [43]. As mentioned above, during endurance exercise C concentrations increase, and previous studies have reported an inhibitory effect of C on T synthesis [9,44] that could interfere with athletes’ recovery and performance, since it participates in protein synthesis and erythropoiesis [45], and could even negatively affect their health due to low bone mineral density and infertility [10].

It has been reported that chronic endurance training may have negative effects on the basal concentration of T, which leads to chronic low levels of this hormone as a consequence of the accumulation of aerobic training over years [46]. In our research, athletes suffered a significant decrease in basal T levels at 3 months accompanied by a significant decrease in LH, a fact that would indicate that there is an alteration of the HPT axis at the central level, decreasing the secretion of GnRH from the hypothalamus that would affect the release of LH and, consequently, the Leydig cells would not be stimulated for T synthesis [47,48]. This could be caused by the fact that in this period the runners trained the highest volume of km per week of the entire season, which forced them to carry out longer training sessions with less recovery between sessions that would promote maintaining high concentrations of C during this phase. MacConnie [48] reported a decrease in LH pulse frequency in highly trained runners. Several studies have reported a negative relationship between T concentrations and high volumes of aerobic training, where the HPT axis is altered in runners who performed more than 100 km/week as occurred with our athletes [49,50]. Flynn et al., observed a decrease in T after the training volume had been increased by 88% for two weeks in swimmers [51].

However, in a recent review suggesting that running mileage alone is not enough to predict the low T concentrations [52], it was proposed that the alterations in the endocrine–reproductive hormonal system observed in endurance runners are related to the development of low energy availability (LEA) [33]. In healthy and active women, it has been established that an adequate energy intake is ≥45 kcal/kg FFM [53]; whether intake in men is similar is currently under debate [52]. In our study, runners reported an energy intake lower than 45 kcal/kg FFM initially and especially at 3 months, a period when the runners ran more km/week, and basal T and LH concentrations were the lowest of the season. De Souza [53] concluded that it is difficult to consume the energy required by athletes who perform chronic strenuous exercise, resulting in an energy deficit that causes alterations in the hypothalamic- pituitary- gonadal axis.

Another factor that could contribute to the decrease in basal T values is the variation in the annual circadian rhythms that this hormone suffers from as a consequence of exposure to the sun, thus, higher peak concentrations have been verified during the summer months, and lower levels during the winter as occurred in our research, since the third month of the study corresponded to the month of January in our region [54]. Low circulating vitamin D concentrations have been associated with a lower total T concentration [55]. Lombardi et al. [56] confirmed the importance of sun exposure and solar irradiance in the vitamin D and T concentrations in professional soccer players during two sports seasons, also, significant correlations between vitamin D and T were reported.

In the second part of the season, at 6 and 9 months, we observed an increase in the basal concentrations of T and LH. It seems that hormonal changes as a consequence of LEA are reversible when the subjects have adequate energy available [57], as occurred in the runners of our study when they performed less km/week and they had adequate energy intake. In addition, the target competitions to be carried out in these periods were shorter distances and higher intensities (maximum 16 min), where the athletes trained less km per week and with greater intensity (more weekly sessions >VT_2_). Previous studies have reported that high-intensity training produces an increase in T [11,58], which could be a consequence of the large reduction in night time C concentrations that occur during these training sessions compared to aerobic training [59]. T could play an essential role in muscle metabolism during the tapering phase [39,60]. T seems to increase the ability of the muscle to refill its glycogen stores through increased activity of muscle glycogen synthetase [61].

This would be very important for adequate regeneration after prolonged exercise and during intensive training periods [62]. Other factors mentioned above are important, such as the annual variation suffered by this hormone; the measures taken at 6 and 9 months correspond to the months of April and July, respectively, where there are more hours of sun exposure and solar irradiance, during those periods which could have favored the increase in vitamin D concentrations and basal T concentrations [63].

As for the I, there was a decrease in its basal values with respect to the initial ones throughout the season, more visible in the periods of greater volume of km trained, also, remarkable negative associations were revealed with the number of km trained per week. Jürimäe, Purge and Jürimäe [64] reported an inverse correlation between I and training volume in elite rowers (r = −0.399, *p* < 0.05). Other study reported a significantly lowered maximal exercise-induced level of I [41]. Reduced insulin levels have been observed in energy-deficient athletes [43].

During prolonged training, the I level decreases because the catecholamine increase inhibits the I secretion [65]. This phenomenon could favor glucose homeostasis with increased glucose availability for the central nervous system [41]. It has been widely reported that training improves insulin sensitivity [66], which can be considered a positive adaptation produced in runners to enhance the use of fatty acids as fuel [23]. Previous studies with athletes have reported a decrease in the plasma I concentration during exercise, followed by an increase during the hours after the end of exercise, in that time there is a decrease in the oxidation of carbohydrates and an increase in the oxidation of fats to favor the repletion of glycogen [22].

The small number of participants and the absence of control of I sensitivity are limitations of the present study. Basal vitamin D concentrations could not be analyzed during the season to observe its circannual rhythm and the possible relationship with the hours of sun and its irradiation. Using Dual-energy X-ray absorptiometry (DXA) or Bioelectrical Impedance Analysis (BIA) are methods that could provide more accurate data on the body composition of subjects (fat mass and fat-free mass) that would have allowed a better assessment of changes in muscle mass and its relationship with dietary intake and hormonal concentrations. Blood samples were not drawn for lactate collection during the treadmill running test.

## 5. Conclusions

Our findings indicate that basal concentrations of T, LH and I in endurance runners are modified throughout the sports season as a consequence of the different training loads, volume and intensity of km they perform per week and energy availability.

In summary, runners who train with a high volume of aerobic km achieve adaptations in the endocrine system, although performing this training with low energy availability causes decreases in basal LH and T concentrations.

## Figures and Tables

**Table 1:** Training characteristics in the athletes during the season.

Training Load	Initial	3 Months	6 Months	9 Months
Total (km/week)	85.71 ± 13.62	105.9 ± 16.85	93.33 ± 14.34	74.76 ± 14.09
>VT_2_ (km/week)	4.29 ± 0.68	12.71 ± 2.02	18.67 ± 2.86	16.45 ± 3.10
<VT_2_ (km/week)	81.43 ± 12.94	93.24 ± 14.83	74.67 ± 11.47	58.31 ± 10.99

VT_2_: anaerobic threshold; >VT_2_: intensity above anaerobic threshold; <VT_2_: intensity below anaerobic threshold.

**Table 2:** Ergospirometric and body composition parameters in the athletes during the season.

Parameters	Initial	3 Months	6 Months	9 Months	η_p_^2^
VO_2_ max (mL/min/kg)	68.12 ± 4.63	67.53 ± 9.54	68.55 ± 6.97	68.60 ± 7.36	0.05
VT_2_ (% VO_2_ max.)	91.02 ± 2.43	92.43 ± 3.59	91.02 ± 3.08	90.96 ± 2.07	0.06
RER	1.05 ± 0.03	1.05 ± 0.04	1.05 ± 0.05	1.04 ± 0.04	0.01
HR maximum	190.4 ± 9.48	193.1 ± 7.80	193.5 ± 9.06	193.8 ± 7.19	0.04
Weight (kg)	65.50 ± 7.30	65.45 ± 7.36	64.67 ± 7.03 *	64.80 ± 7.34 *	0.07
Fat mass (%)	8.18 ± 1.04	8.23 ± 1.04	8.19 ± 1.29	8.21 ± 1.07	0.04
Fat-free mass (kg)	60.15 ± 6.70	60.07 ± 6.75	59.38 ± 6.45	59.48 ± 6.73	0.02
∑6 skinfold (mm)	45.65 ± 10.88	46.49 ± 10.69	44.92 ± 8.16	45.37 ± 9.11	0.03

VO_2_ max: maximum oxygen uptake; VT_2_: ventilatory anaerobic threshold; HR maximum: heart rate maximum; ∑6: sum of 6 skinfolds * *p* < 0.05 initial vs. 3/6/9 months; η_p_^2^: partial eta squared.

**Table 3:** Nutritional intake.

Parameters	Initial	3 Months	6 Months	9 Months
Energy (kcal/d)	2855.21 ± 511.32	2515.48 ± 427.18	2902.37 ± 522.62	3108.78 ± 770.12
EA (kcal/kg/FFM/d)	43.58 ± 4.32	41.87 ± 3.15	48.88 ± 5.63	52.26 ± 4.87
HC (g/kg/d)	5.26 ± 1.21	5.18 ± 1.14	6.25 ± 1.38	6.13 ± 1.50
Proteins (g/kg/d)	1.73 ± 0.79	1.69 ± 0.35	1.85 ± 0.53	1.89 ± 0.63
Lipids (g/kg/d)	1.78 ± 0.40	1.63 ± 0.28	1.58 ± 0.52	1.72 ± 0.74

HC: carbohydrates; FFM: fat-free mass; EA: energy availability.

**Table 4:** Hormonal changes during the season.

Parameters	Initial	3 Months	6 Months	9 Months	η_p_^2^
Insulin (μIU/mL)	10.25 ± 7.99	7.81 ± 6.15 **	7.62 ± 5.50 **	9.89 ± 5.32 *	0.51
LH (mIU/mL)	8.85 ± 4.10	6.30 ± 2.86 **	7.59 ± 2.32 ^#^	7.95 ± 3.49 ^#^	0.29
Testosterone (ng/mL)	6.59 ± 0.92	5.83 ± 1.10 **	6.72 ± 0.94 ^#^	7.01 ± 1.50 ^#^	0.32
Cortisol (ng/mL)	89.26 ± 21.85	91.41 ± 27.32	98.06 ± 31.28	103.9 ± 38.04	0.05
T/C	0.07 ± 0.01	0.06 ± 0.02	0.07 ± 0.02	0.07 ± 0.02	0.03

LH: luteinizing hormone; T: testosterone; C: cortisol; * *p* < 0.05 differences between initial vs. 3/6/9 months; ** *p* < 0.01 differences between initial vs. 3/6/9 months; # *p* < 0.05 differences between 3 months vs. 6/9 months; η_p_^2^: partial eta squared.

**Table 5:** Simple linear regression between the plasmatic concentration of hormones and total km trained.

Hormones	β (95% CI)	SE	*r*	*R^2^*	*p*
Insulin	−0.577 (−0.984/0.030)	0.255	0.363	0.142	0.017
LH	−1.105 (−2.769/−0.440)	0.585	0.113	0.025	0.302
Testosterone	−2.145 (−5.247/−3.543)	1.109	0.131	0.028	0.213
Cortisol	0.310 (−0.124/0.544)	0.167	0.202	0.075	0.043

LH: luteinizing hormone; β: beta coefficient; SE: standard error; CI: confidence interval; R^2^: coefficient of determination; r: Pearson’s coefficient of correlation; *p*: *p* value.

**Table 6:** Simple linear regression between the plasmatic concentration of hormones and km trained with intensity higher than VT_2_.

Hormones	β (95% CI)	SE	*r*	*R* ^2^	*p*
Insulin	−0.442 (−0.579/−0.306)	0.069	0.580	0.336	0.000
LH	−0.320 (−0.707/0.068)	0.195	0.178	0.032	0.104
Testosterone	0.276 (−0.812/1.364)	3.638	0.056	0.003	0.615
Cortisol	0.238 (−0.004/0.081)	2.150	0.193	0.037	0.079

LH: luteinizing hormone; β: beta coefficient; SE: standard error; CI: confidence interval; R^2^: coefficient of determination; r: Pearson’s coefficient of correlation; *p*: *p* value.
